# Medical Items

**Published:** 1894-04

**Authors:** 


### *MEDICAL ITEMS.*

---

Dr. Edward E. Kerr, of Chattanooga, was recently expelled from the Chattanooga Medical Society for publicly indorsing the Keely cure. The Code of Ethics speaks in no uncertain tone in regard to our relation to patent and secret remedies. We think, therefore, the above violation of the Code met with a very just reward.

---

At the March examinations positions on the Grady Hospital House Staff were awarded as follows: Dr. M. G. Campbell, House Physician and Surgeon; Dr. O. H. Weaver, First Assistant; Dr. J. B. Diamond, Second Assistant; Dr. W. S. Little, Third Assistant. Dr. Diamond received second mark, but chose third assistant's place, for a longer term.

---

The following anecdote is going through the journals: A gentleman walked into a drug store and asked: "Are you a pharmacist?" "Yes." "Registered?" "Yes." "Is that your diploma over there?" "Yes." "Well, I guess you can give me a pound of borax." And this reminds us of a late occurrence in this ctiy. One of the vaccinating corps called at a certain place and the lady of the house asked: "Are you a doctor?" "Yes." "Regular graduate?" "Yes." "From what school?" "——— Medical College." "How many years have you practiced?" "Four." "Well, I'll get you to vaccinate my cook."

---

The fourth annual meeting of "The Association of Military Surgeons of the United States," will be held in Washington, D. C., May 1st, 2d and 3d, 1894.

This National Organization is composed of Medical Officers of the United States Army, United States Navy, National Guard of the United States and the Hospital Marine Service—in whose service are many of the most celebrated and distinguished surgeons of our country. A brilliant and able literary programme will be presented. The afternoon of one day will be set apart for an object lesson from the "Manual of Drill," by the Hospital Corps. The evenings will be given up to social entertainments. The President, Colonel Nicholas Senn, of Chicago, will deliver an address on "Abdominal Surgery on the Battle-Field." Many other interesting papers will be read on military surgery. Those who wish to attend the meeting should notify Major George Henderson, Chairman Committee of arrangements, 817 T St., N. W., Washington, D. C.

---

Dr. E. C. Angell, representing the *Sanitarian*, was one of the party of medical editors lately in our midst. Concerning his visit to the South he says:

Every one of the places we have visited had attractions and entertainments that would need a month's time to fully comprehend and describe. I am more than grateful for the opportunity to visit these several Southern cities, and to have met the business and professional men who have established their success.

I have been favorably impressed with the ability, the fraternal greeting, the courtly and kindly urbanity of the physicians in whose hands rest the health and life of the citizens. They are eminently fitted to inspire confidence and hope in those who entrust themselves to their care.

Preventive medicine calls loudly for natural remedies. Are not these remedies too much ignored, too little understood in these Southern cities? It would be impossible to find as many cities in the North or West that are so deficient in appliances for the employment of natural remedies.

Sanitariums are indispensable requisites to the best results; and I greatly fear that their chief obstacle is the blind belief of some of our Southern brothers in the superior efficacy of drugs.

From wide observation at home and abroad, it is my impression that it would be difficult to find among any other people, otherwise so highly cultured, communities of equal size of those we have visited so deficient in sanitary appliances necessary to the maintenance of health resorts.

